# Leading for the long haul: a mixed-method evaluation of the Sustainment Leadership Scale (SLS)

**DOI:** 10.1186/s13012-018-0710-4

**Published:** 2018-01-19

**Authors:** Mark G. Ehrhart, Elisa M. Torres, Amy E. Green, Elise M. Trott, Cathleen E. Willging, Joanna C. Moullin, Gregory A. Aarons

**Affiliations:** 1 Department of Psychology, University of Central Florida, Orlando, FL USA; 2 Center for Organizational Research on Implementation and Leadership (CORIL), San Diego, CA USA; 3 Department of Psychology, George Mason University, Fairfax, VA USA; 4 Department of Psychiatry, University of California, San Diego, 9500 Gilman Dr. (0812), La Jolla, CA 92093-0812 USA; 5 Child and Adolescent Services Research Center (CASRC), San Diego, CA USA; 6 Pacific Institute for Research and Evaluation, Behavioral Health Research Center, Albuquerque, NM USA; 7 Department of Anthropology, University of New Mexico, Albuquerque, NM USA; 8 School of Pharmacy and Biomedical Science, Faculty of Health Sciences, Curtin University, Perth, Western Australia

## Abstract

**Background:**

Despite our progress in understanding the organizational context for implementation and specifically the role of leadership in implementation, its role in sustainment has received little attention. This paper took a mixed-method approach to examine leadership during the sustainment phase of the Exploration, Preparation, Implementation, Sustainment (EPIS) framework. Utilizing the Implementation Leadership Scale as a foundation, we sought to develop a short, practical measure of sustainment leadership that can be used for both applied and research purposes.

**Methods:**

Data for this study were collected as a part of a larger mixed-method study of evidence-based intervention, SafeCare®, sustainment. Quantitative data were collected from 157 providers using web-based surveys. Confirmatory factor analysis was used to examine the factor structure of the Sustainment Leadership Scale (SLS). Qualitative data were collected from 95 providers who participated in one of 15 focus groups. A framework approach guided qualitative data analysis. Mixed-method integration was also utilized to examine convergence of quantitative and qualitative findings.

**Results:**

Confirmatory factor analysis supported the a priori higher order factor structure of the SLS with subscales indicating a single higher order sustainment leadership factor. The SLS demonstrated excellent internal consistency reliability. Qualitative analyses offered support for the dimensions of sustainment leadership captured by the quantitative measure, in addition to uncovering a fifth possible factor, available leadership.

**Conclusions:**

This study found qualitative and quantitative support for the pragmatic SLS measure. The SLS can be used for assessing leadership of first-level leaders to understand how staff perceive leadership during sustainment and to suggest areas where leaders could direct more attention in order to increase the likelihood that EBIs are institutionalized into the normal functioning of the organization.

## Background

There is growing interest in examining how and what aspects of organizational context impact whether service providers use evidence-based interventions (EBI), treatments found to produce positive outcomes in rigorous research studies [1], with their clients. A number of implementation frameworks (such as the Exploration, Preparation, Implementation, and Sustainment (EPIS) framework [2] and the Consolidated Framework for Implementation Research (CFIR) [3]) highlight organizational characteristics and processes comprising the inner organizational context of EBI implementation. Factors include organizational culture, climate, structure, size, and readiness for change. In line with these frameworks, research across a number of health and human service settings (e.g., hospitals, mental health, substance use disorder treatment, child welfare) points to the critical role of the inner context for implementation (e.g., [4–8]). For example, the extent to which leadership and organizational environments foster team learning in surgical settings is associated with implementation success [9, 10].

A long history of research on organizational culture and climate has identified leadership as a critical facilitator of the context that develops in organizational settings (see review by Ehrhart, Schneider, and Macey [4]). Although there is little consensus in the literature on the exact distinction between leadership and management [11], we use the term leadership to be consistent with related literature in this area and to capture the influence those in a leadership role have on the units they lead. Thus, for this study, we adopt Yukl’s [11] definition of leadership as “the process of influencing others to understand and agree about what needs to be done and how to do it, and the process of facilitating individual and collective efforts to accomplish shared objectives” (p. 7).

With regard to EBI implementation, the role of leaders across different levels has been emphasized in multiple implementation frameworks (e.g., EPIS [2] and CFIR [3]). Despite the need for more empirical studies of leadership in implementation [12, 13], recent research has linked a variety of leadership behaviors with implementation outcomes (e.g., [14–19]). Although much of this research has focused on general leadership behaviors, Aarons, Ehrhart, and Farahnak [20] have proposed that leadership behaviors focused on implementation may be more proximal predictors of implementation-related outcomes. Their development of the Implementation Leadership Scale (ILS) forms the foundation for the current paper’s focus on leadership for sustainment.

The development of the ILS was based, in part, on research literature from business/management and industrial/organizational psychology on leaders’ use of “embedding mechanisms” (i.e., actions that leaders can take to communicate their core values) to develop or shape organizational culture and climate [21] and strategically focused leadership as a predictor of strategic climates and subsequent outcomes [22–24]. As supported by theory and empirical research on service climate [23] as well safety climate [25], strategically focused leadership is proposed to be a primary predictor of strategic climates, which are strong predictors of employees’ motivation and behavior in support of the achievement of the unit’s goals related to the specific strategic imperative. These behaviors are then the proximal predictors of the unit’s strategic outcomes, such as accidents in safety research [25] or customer satisfaction in the service literature [23].

To develop implementation leadership items, Aarons et al. [20] drew from other measures of strategically focused leadership [23, 25], Schein’s [21] embedding mechanisms (e.g., what leaders pay attention to and how they react to organizational crises), and implementation subject matter experts with the goal of identifying those aspects of the leadership of first-level supervisors that are most relevant for influencing their subordinates during implementation efforts. After a process of narrowing the item pool and dimensions through exploratory factor analysis, they identified four dimensions of implementation leadership: proactive leadership (i.e., the degree to which the leader establishes clear goals and plans, and removes obstacles during implementation), knowledgeable leadership (i.e., the degree to which the leader is knowledgeable about EBIs), supportive leadership (i.e., the degree to which a leader supports staff efforts to use and learn EBIs, and recognizes their efforts in doing so), and perseverant leadership (i.e., the degree to which the leader persists and moves forward in the implementation process despite problems and challenges).

Despite progress in understanding the organizational context for implementation and specifically the role of leadership in implementation, its role in sustainment has received almost no attention. Sustainment has been defined as the continued use of an EBI over time with fidelity and ongoing support such that the desired benefits of the EBI are maintained [13, 26]. In general, factors affecting the long-term sustainment of EBIs are understudied relative to those influencing implementation [13, 27]. Measures asking about leadership for implementation may be less relevant in organizations that have moved to the sustainment phase. In addition, there may be similarities and differences between implementation and sustainment that create some ambiguity about whether the specific dimensions of leadership that are critical during implementation are also critical during sustainment. For instance, whereas implementation involves establishing new practices in an organization, sustainment is focused on continuing those practices over time. Both implementation and sustainment involve ongoing organizational learning to be successful [28], but sustainment is, by definition, dependent on implementation since implementation processes come first. Thus, if implementation is managed poorly, then sustainment will likely have little chance of success [29]. Although many of the same organizational characteristics that lead to successful implementation are also likely to contribute to successful sustainment [29], the two processes may have both common and unique aspects requiring distinct leadership approaches.

The purpose of this study is to contribute to our understanding of EBI sustainment by employing mixed-method research to examine a measure of sustainment leadership, the Sustainment Leadership Scale (SLS). We define sustainment leadership as the attributes and behaviors of leaders that support the effective sustainment of EBI implementation. In light of the arguments to be made for both the overlap and distinctiveness of implementation leadership and sustainment leadership, we used a research design that addresses both. Specifically, we first used a quantitative approach to test whether the dimensions of the ILS hold in the context of sustainment. We next used a qualitative design to connect descriptions of leadership behaviors during sustainment to that same dimensional structure, and then to address the possibility of differences in critical leadership behaviors in implementation and sustainment phases, we identified any new themes/dimensions in the qualitative study that did not align with the original proposed SLS dimensions. In addition, although the ILS was originally developed with a focus on the leadership behaviors of first-level supervisors (which we follow in our quantitative analyses), qualitative methods allowed for frontline providers to comment on leadership of both their first-level supervisors and upper-level leaders in their organization.

This research addresses multiple gaps in the implementation literature. First, we contribute to the general knowledge on sustainment of EBIs, which has been understudied in the implementation literature [13, 27, 30]. Second, we specifically address the role of leadership in sustainment and clarify whether the behaviors that are critical to implementation are also viewed as crucial to sustainment. Finally, we contribute to the limited amount of validated measures assessing various components of sustainment [13] by developing a short, practical measure of sustainment leadership. Such a measure should be useful for researchers with a specific interest in the role of leadership and the organizational context on EBI post-implementation, and for practitioners interested in what leaders can do to contribute to the sustainment of EBI use over time.

## Methods

### Study context

This research was part of a larger mixed-method study of evidence-based intervention (EBI) sustainment funded by the U.S. National Institute of Mental Health (NIMH R01MH072961). The sustainment study is an extension of previous studies that examined the implementation of SafeCare®, a home-based, behavioral and psychosocial EBI developed to reduce child neglect and promote child health and development. SafeCare is comprised of three modules including health, home safety, and parent-child or parent-infant interaction. Home visitors (referred to from this point onward as providers) are directly involved in the delivery of SafeCare services. Providers are trained to administer the three modules through assigned homework, role-play, and hands-on demonstrations. Coaches function as the fidelity support and monitoring system for providers by providing expertise in both implementing SafeCare and ongoing SafeCare fidelity assessment and feedback [31]. The role of coaches is distinct from that of frontline supervisors, who oversee the case and well-being of the family and are often involved in the administrative oversight of their team. For the current study, 30% of the participating agencies employed a frontline supervisor who was also trained as a certified SafeCare coach. In these cases, the individual served a dual role as administrative supervisor and coach in the SafeCare model. In the remaining 70% of agencies, the frontline leader provided administrative and clinical supervision; however, ongoing coaching and fidelity monitoring was conducted by a separate trained staff member with SafeCare expertise. The EBI sustainment study examines various factors in the outer context (i.e., outside the agency or organization, such as the service environment) and the inner context (i.e., internal to the agency or organization, such as its sustainment climate) that relate to sustainment.

The setting for this study included seven separate child welfare systems. Included were one statewide system referred to here as “State A” and six county-wide systems in a state referred to as “State B.” Training in SafeCare began between 1.5 and 10 years prior to the commencement of this data collection (two systems for 1.5 years, one for 2.5 years, three for 3.5 years, and one for 10 years). According to the 2010 U.S. Census Bureau, State A had a population of approximately 3.7 million residents, almost 42% of whom lived in rural areas. In State A, SafeCare was implemented through a state-operated child welfare system with all services guided, contracted, and funded by the state government. State B included four primarily urban and two primarily rural counties involved in implementing SafeCare, ranging in population from just over 180,000 to approximately 2.3 million residents.

### Quantitative data

#### Participants

Survey data from 2014 were utilized in this study. Participants were 157 providers working in 31 teams across 22 community-based organizations (i.e., agencies) within the 7 child welfare systems. In the context of this study, provider refers to home visitors who are trained in the SafeCare model. The response rate was 95%. Participant mean age was 38.33 years (*SD* = 11.60; range = 22 to 71) and the majority of respondents were female (91.1%). The racial/ethnic distribution of the sample was 57% Caucasian, 10.8% African American, 4.4% Asian American or Pacific Islander, 5.1% Native American, 6.3% multiple races, and 16.5% “other.” A total of 38% identified as being of Hispanic/Latino origin. With regard to education, 60.8% of participants held bachelor’s degrees, 16.5% held master’s degrees, 11.4% had some college experience, 8.9% indicated some graduate work, and 1.9% held high school diplomas.

#### Procedure

The research team made initial contact with an agency executive at each site. Providers were then contacted via email for recruitment in the study. Each participant was emailed an invitation to participate including a unique username and password, as well as a link to the survey. Upon accessing the survey though their unique codes, participants were then prompted to review the informed consent. After agreeing to participate, participants were presented with the questions in the survey, with the ability to pause and resume at any time. The online survey took approximately 45 to 90 min to complete, and upon completion, participants received incentive vouchers ($30).

#### Measure

The Sustainment Leadership Scale (SLS) was adapted from the ILS in two ways. First, all references to “implementation” were changed to “sustainment.” The measure instructions to participants described sustainment as “continued use and support for SafeCare by your organization.” Second, the measure was adapted to specifically refer to the EBI being implemented, SafeCare. Participants completed ratings of their supervisor’s sustainment leadership behaviors. Supervisors were frontline leaders who oversaw their team’s delivery of the SafeCare model. The SLS included 12 items scored on a 0 (“not at all”) to 4 (“to a very great extent”) scale. There were four subscales: proactive leadership (*α* = .94), knowledgeable leadership (*α* = .98), supportive leadership (*α* = .96), and perseverant leadership (*α* = .94). The total SLS score (*α* = .97) was created by computing the mean of the four subscales.

#### Statistical analysis

The SLS factor structure was tested using confirmatory factor analysis (CFA) conducted in Mplus statistical software [32], adjusting for the nested data structure (clinicians nested in 31 teams). Mplus uses maximum likelihood estimation with robust standard errors (MLR), which appropriately adjusts standard errors and chi-square values. Our data contained no missing values. To examine multivariate outliers, we examined Mahalanobis distance. A total of 10 multivariate outliers were identified. A follow-up sensitivity analysis, removing the 10 outliers resulted in comparable model fit statistics. Thus, we report the results for the model with all data. In order to determine model fit, several empirically supported indices were assessed: the comparative fit index (CFI), the root mean square error of approximation (RMSEA), and the standardized root mean square residual (SRMR). The CFI values greater than .95, RMSEA values less than .08, and the SRMR values less than .06 indicate acceptable model fit [33]. Consistent with the ILS development study [20] from which this measure was adapted, the higher-order model was tested to evaluate the four-factor model with each subscale as an indicator of the overall sustainment leadership latent construct. Internal consistency reliability of each subscale and the total scale using Cronbach’s alpha were examined. Aggregation analyses were also conducted to examine the amount of dependency among observations within groups.

### Qualitative data

#### Participants

Qualitative data were collected from SafeCare providers (*N* = 95) who participated in one of 15 focus groups. The focus groups consisted of an average of six participants per group. Focus groups were specific to each agency and conducted separately with coaches and providers. The composition of the focus groups was determined by the agency leadership based on provider availability. Two thirds of the focus group participants also completed the quantitative survey. Participants in the focus groups were primarily female (87.4%) and had an average age of 37.93 (SD = 12.06; range = 23 to 68). The highest reported education level was bachelor’s degree (64.9%), master’s degree (20.2%), some graduate work (7.4%), some college (5.3%), and high school (2.1%). Of the focus group participants, the majority were Caucasian (56.8%), followed by those identifying as “other” (22.1%), African American (8.4%), Asian American or Pacific Islander (7.4%), and Native American (5.3%). Hispanic ethnicity was reported by 37.9% of the participants.

#### Procedure

The research team made initial contact with agency executives participating in the ongoing study of SafeCare sustainment to set up a time to conduct focus groups with providers. For teams with eight or fewer providers, the entire team was invited to participate in the focus groups. For teams with more than eight providers, the supervisor was asked to select a sample of six to eight providers who could be available during the selected time frame. Prior to each focus group, the research team described the purpose of the focus group and obtained informed consent from participants. Two anthropologists conducted focus groups with providers from each of the seven child welfare service systems implementing SafeCare between 2012 and 2013, one and a half to 10 years post initial SafeCare training and implementation. Focus groups were typically 90 min in length and focused on perceptions regarding SafeCare sustainment. Semi-structured questions addressed positive and negative influences on SafeCare implementation and sustainment. The term “sustainment” was not used in the instructions in order to use terminology that was accessible and understandable to participants who may be unfamiliar with the concept of EBI sustainment. Instructions for the qualitative data collection stated that the purposes were to understand “what’s working with SafeCare, the challenges involved in delivering this program, and what it will take to keep this service available in the child welfare system in future years.” Thus, the focus was on the current status of the delivery of the EBI and its ongoing delivery in the future. All of the teams participating were in the sustainment phase and had been sustaining SafeCare between 1.5 and 10 years prior to the commencement of this data collection.

The main questions analyzed included: “How have *leaders* within your team or agency supported use of SafeCare? Please specify whether you are referring to a team or agency leader.” A probe sought to further clarify the role of leadership in sustaining SafeCare, “What have they (e.g., your leader[s]) done to potentially undermine the use of SafeCare?” Leaders within this context refer to frontline team leaders as well as other agency leaders, including program managers, area directors, and executive directors.

#### Data preparation and analysis

All focus groups were digitally recorded, professionally transcribed, and checked for accuracy by at least one author. NVivo 10 qualitative data analysis software facilitated data management and analysis [34]. Coding for this study focused on a set of questions that explicitly asked about leadership rather than the full transcript. Three of the authors (MGE, AEG, and GAA) implemented a framework approach to guide the data analysis process [35]. This largely deductive methodology used the SLS constructs to link the qualitative findings to the quantitative study, while facilitating the inductive emergence of new themes. First, researchers familiarized themselves with the transcripts ensuring the constructs of the SLS represented a suitable thematic framework. Segments of text ranging from a phrase to several paragraphs were then assigned to the SLS codes. The codes were based a priori on the original dimensions of sustainment leadership (proactive leadership, knowledgeable leadership, supportive leadership, and perseverant leadership), including the definitions of these dimensions and the items used to measure them in the quantitative measure. Note that because the scales each had only three items, the process was generally more focused on whether the qualitative data fell within the definition of the dimensions rather than corresponding to specific SLS item content. This process enabled examination of both the salience (i.e., the proportion of codes within each dimension) and meaning of these dimensions for providers through the provision of descriptive data based in the actual words of participants and directly reflecting their own perceptions and experiences ([36], p. 456). As the iterative process of coding progressed, inductive open coding of the data located new themes and issues related to leadership that did not fit within the SLS constructs [37]. As such, the process allowed both confirmation and refinement of the framework (SLS constructs). In this approach to analysis, each author coded sets of transcripts and created detailed memos that both described and linked codes. The authors then shared and discussed their work as a group in order to reach a consensus on a final set of codes.

### Mixed-method analysis

For the purpose of this paper, we conducted a mixed-method analysis seeking triangulation, a process for strategically utilizing multiple methods together, in order to examine convergence of quantitative and qualitative datasets [38–40]. We first examined whether our data resulted in the same or similar themes (i.e., convergence), mapping the themes that emerged from the focus groups to the quantitative dimensions. We also examined if our qualitative data provided further insight into the SLS construct than what were furnished by the quantitative data (i.e., expansion). We acknowledge that our design deviates from the traditional sequential-exploratory mixed-method approach to instrument development in which the research topic is first explored qualitatively with participants, with qualitative findings then used to develop items for quantitative psychometric testing [39]. Our study, however, employed a different approach as our first goal was to determine whether an existing measure of implementation leadership could be modified to measure sustainment leadership using the same factor structure. Our secondary goal was then to use the qualitative data to further validate the dimension of sustainment leadership with a group of service providers sustaining an EBP and to expand upon whether additional themes related to sustainment leadership emerged.

## Results

### Quantitative results

Aggregation statistics (ICC(1) and *a*_*wg*(*j*)_) were calculated to explore whether sustainment leadership could be considered a unit-level construct in this sample. The SLS had an ICC(1) value of .05, and an average *a*_*wg*(*j*)_ value of .73 (range of .56 to .95). According to LeBreton and Senter (2008) [41], the ICC(1) value indicates a small-to-medium effect size for the group, and the mean *a*_*wg*(*j*)_ value indicates strong within-group agreement, suggesting limited between-group variability. Although these statistics provide acceptable minimum support for aggregation to the unit level, there are additional implications addressed in the “Discussion” section.

The SLS data provided good model fit for the second-order factor model of the ILS (*χ*^2^(50) = 94.26, *p* < 0.001; CFI = .97, RMSEA = .075; SRMR = .039). Table 1 provides the standardized factor loadings for the items on each first-order factor, which ranged from .85 to .98, and the loadings of each dimension on the second-order factor, which ranged from .87 to .94. All the loadings in the model were significant at *p* < .001. The table also shows the SLS scale reliabilities and item means and SDs. Cronbach’s alphas for the subscales ranged from .94 to .98 and was .97 for the overall scale, demonstrating excellent internal consistency reliability. The SLS factors provided the following interfactor correlations: proactive leadership with knowledgeable leadership (*r* = .74, *p* < .001), supportive leadership (*r* = .79, *p* < .001), and perseverant leadership (*r* = .88, *p* < .001); knowledgeable leadership with supportive leadership (*r* = .79, *p* < .001), and perseverant leadership *r* = .78, *p* < .001); and supportive leadership with perseverant leadership (*r* = .84, *p* < .001).

**Table 1:** Sustainment Leadership Scale, subscale and item statistics for 2014 data

SLS items, subscales, and total	Mean	*sd*	*α*	CFA factor loadings
1. Proactive leadership	2.80	1.11	.94	.93
Developed a plan to facilitate sustainment of SafeCare	2.82	1.16		.96
Removed obstacles to the sustainment of SafeCare	2.65	1.22		.87
Established clear department standards for the sustainment of SafeCare	2.93	1.14		.93
2. Knowledgeable leadership	3.11	1.00	.98	.84
Is knowledgeable about SafeCare	3.13	0.99		.97
Is able to answer staff questions about SafeCare	3.09	1.03		.98
Knows what he/she is taking about when it comes to SafeCare	3.12	1.03		.98
3. Supportive leadership	3.23	0.93	.96	.90
Recognizes and appreciates employee efforts toward successful sustainment of SafeCare	3.18	1.00		.91
Supports employee efforts to learn more about SafeCare	3.21	0.99		.99
Supports employee efforts to use SafeCare	3.29	0.92		.96
4. Perseverant leadership	3.05	0.95	.94	.94
Perseveres through the ups and downs of sustainment of SafeCare	3.06	0.95		.97
Carries on through the challenges of sustaining SafeCare	3.10	0.91		.98
Reacts to critical issues regarding the sustainment of SafeCare by effectively addressing the problem(s)	2.97	1.13		.86
Sustainment Leadership Scale total	3.05	0.92	.97	

Note: *N* = 157 for means, standard deviations, ICC, and alphas; *sd* = standard deviation; factor loadings are standardized

### Qualitative results

The analysis of the qualitative data from the focus group transcripts offered support for the dimensions of sustainment leadership captured by the quantitative measure (i.e., Proactive, Knowledgeable, Supportive, and Perseverant). A fifth factor emerged from the qualitative interviews that was not previously identified in the quantitative SLS: available leadership. Table 2 provides the Sustainment Leadership Scale items side-by-side with the qualitative results supporting convergence with the four dimensions of sustainment leadership across methods. Expansion was demonstrated in the emergence of a newly identified dimension representing availability of leadership. This emerged from providers’ descriptions of the usefulness of their leader being available and accessible as a factor that contributed to their team/agency successfully sustaining SafeCare. It should also be noted that although interview questions cited general leadership across levels in the organization, almost all comments focused on the leadership of first-level supervisors. Thus, the findings below generally refer to first-level supervisors except where we specifically note that the participants were describing higher-level leaders.

**Table 2:** Integration of mixed-method results (quantitative items and qualitative themes) demonstrating convergence and expansion of findings

	Type of Method		
Dimension	Quantitative Items	Qualitative Findings	MM Function
Proactive	- Developed a plan to facilitate sustainment of SafeCare - Removed obstacles to the sustainment of SafeCare - Established clear department standards for the sustainment of SafeCare	- Leaders were described as proactively communicating information about SafeCare. - Providers mentioned their leaders developing processes to ensure SafeCare is employed - Leaders proactive in ensuring providers have enough referrals to employ SafeCare.	Convergence
Knowledgeable	- Is knowledgeable about SafeCare - Is able to answer staff questions about SafeCare - Knows what he/she is taking about when it comes to SafeCare	- Leaders were described as knowing SafeCare in and out, and able to quickly direct providers to resources. - Leaders provide providers SafeCare advice to questions they have with clients. - Providers described leaders as having extensive past experience delivering SafeCare, and understand its processes.	Convergence
Supportive	- Recognizes and appreciates employee efforts towards successful sustainment of SafeCare - Supports employee efforts to learn more about SafeCare - Supports employee efforts to use SafeCare	- Leaders were described as being very supportive to providers, encouraging them to excel in their delivery of SafeCare. - Leaders were described as ‘cheerleaders’ - Providers mentioned that their leaders solicited their input regarding SafeCare and were open to answer their questions.	Convergence
Perseverant	- Perseveres through the ups and downs of sustainment of SafeCare - Carries on through the challenges of sustaining SafeCare - Reacts to critical issues regarding the sustainment of SafeCare by effectively addressing the problem(s)	- When there are issues or something is going wrong with SafeCare, leaders meet with providers to work through the problems. - Providers described their leaders as using creative strategies to address ongoing referral problems.	Convergence
Available	*Not applicable*	- Providers reported that though their leaders were busy, they were always available. - Leaders were mentioned as being easily accessible, taking the time to meet if there were concerns or issues.	Expansion

#### Proactive leadership

Although not the most salient construct, providers still identified leaders taking initiative to resolve issues that potentially threatened sustainment of SafeCare as an important component. Providers offered multiple examples of their leaders employing proactive leadership, describing them as reminding staff of the process of SafeCare and following up with staff to ensure that SafeCare continued to be used with clients. For example, one provider mentioned that his/her leader “…does put that emphasis on doing what’s important [for SafeCare] and what the main issue is first, and then she [supervisor] really makes sure she pays attention to that and makes sure we’re getting it as well.” Similarly, a second provider reported how his/her leader established road maps in which plans to use SafeCare with clients were laid out in detail. Providers at one agency observed that upper management worked to develop a mission statement that incorporated an organizational commitment to and expectations for the SafeCare program. Favorably viewed leaders were also characterized as actively encouraging the use of SafeCare to system stakeholders employed outside of their agencies, asking important questions such as, “Have you looked at SafeCare?” to Child Welfare Service (CWS) workers who make referral decisions for services. One provider praised their leader for attending CWS staff meetings to help identify cases suitable for a SafeCare referral, while a second provider lamented the lack of proactivity from their leader, observing that “Our supervisor doesn’t really say anything much about SafeCare.”

#### Knowledgeable leadership

Providers made several comments regarding their leaders’ knowledge about SafeCare and its intricacies. Some providers commented generally about the length of time their leader has been working in the field of child welfare services. One individual explained, “She knows it [SafeCare] works. Because she used to be a [case manager] and now she’s a supervisor, so she’s seen these positive changes in families [because of the intervention].” Others were more specific about their leaders’ extensive knowledge in helping them continue to utilize SafeCare, commenting “They [supervisors] both gave me some really good ideas in trying to modify the module and to get the information needed to the family.” Leaders were described as being able to answer staff’s questions about SafeCare processes, and providing suggestions on how to maintain fidelity. Providers mentioned that their leaders were knowledgeable about the importance of research-based practices and emphasized their value to staff. Another agency director was praised for “know[ing] SafeCare in and out.”

There were also negative views of leaders’ knowledge of SafeCare, as some providers suggested that their leaders were too far removed from practice and generally unaware of the issues that arise when implementing SafeCare. However, the majority of these providers mentioned that this lack of understanding of the intricacies of SafeCare applied to higher-level managers and directors at their agencies rather than to their direct frontline supervisors.

#### Supportive leadership

There was a general consensus among providers on the critical need and value of support from their leaders. Providers offered several examples of how their leaders supported them and the practice of SafeCare. One group of providers mentioned that their program manager supported them by being flexible with their paperwork and understanding that they sometimes needed to prioritize some tasks over others. Another provider noted that the agency director was supportive and understanding of his/her role as a parent, facilitating a work schedule that enhanced his/her ability to deliver SafeCare. Many providers affirmed that their leaders were behind them at every step, while also providing suggestions to enhance their SafeCare practice. One provider discussed going on a ride along with their frontline supervisor, who then observed their work and provided feedback into making delivery of SafeCare easier. Leaders were described as seeking input from the providers, making sure that all were supportive of SafeCare. Providers also mentioned how leaders provided various words of encouragement, with a provider even describing their leader as a “cheerleader for SafeCare.”

#### Perseverant leadership

In line with the definition of this dimension, responses were coded as perseverant leadership if they described actions taken in response to a specific problem or challenge. Leaders were identified as being responsive and moving forward when troubleshooting issues came up by working actively to advance solutions. For example, one team of providers discussed a leader’s problem solving in reaction to a significant drop in the number of SafeCare referrals—a drop that limited their organization’s ability to sustain SafeCare. As one team member noted, “I think she’s really made those attempts to get CWS to refer us more cases. She really wants us to have a SafeCare program.” A second team member discussed their leader’s creative problem solving by pursuing multiple avenues to increase the number of SafeCare cases assigned to her providers: “she has been creative on looking to other—not just CWS, but maybe in the agency—to see how can we open other cases because we don’t have that many.” Note that although such actions could be coded as proactive leadership, when those actions were taken in response to problems with getting enough cases, they were coded as perseverant leadership. Providers also commented on how their leaders helped them with specific challenges related to delivering SafeCare. One provider detailed a situation in which they were having issues with delivering a SafeCare module due to the age of the child. The leader openly discussed with the provider the issues and suggested ideas on how to modify the module to ensure the necessary information was getting to the child’s family. There were also examples of low levels of perseverant leadership, such as when a supervisor was described as always handing off any issues with SafeCare to the coach. The provider noted that “if there’s something challenging… she will ask me, ‘Do I need [the coach] to come out there?’ It’s really only [the coach]. I don’t think the supervisors think that’s their job or in their job description.”

#### Available leadership

There was a set of responses from providers regarding their leader’s availability, specifically with regard to availability to help answer questions and resolve issues relating to SafeCare sustainment. Some providers mentioned that having their frontline supervisors in close proximity was helpful as they were able to ask questions in person. Furthermore, providers mentioned that even though their leaders were busy with other responsibilities, they always made the time to assist them in answering questions relating to SafeCare, “no matter whom they are [with] or where they’re at.” Available leadership was also discussed by one provider as occurring at multiple levels to address any concerns, “I feel that if we had a problem or concern about anything that we could contact any of those people [leadership staff], from the director all the way down.”

## Discussion

The goal of this research was to extend the concept of implementation leadership to sustainment leadership using both quantitative and qualitative methods, thus expanding our understanding of how leaders “lead for the long haul.” We adapted a measure of implementation leadership (the ILS) to represent sustainment leadership (i.e., the Sustainment Leadership Scale). We also analyzed qualitative data on sustainment leadership to provide further support for the generalizability of the ILS dimensions to sustainment and to explore the possibility that additional dimensions of leadership may manifest during sustainment. Analyses of the quantitative data indicated strong support for the adapted measure. These results suggest that the aspects of leadership that research has shown to be critical during implementation (being proactive, knowledgeable, supportive, and perseverant) are also highly relevant to sustainment. This finding is consistent with the suggestion by Scheirer [29] that the organizational issues factoring into successful implementation likely resemble those needed for successful sustainment. Thus, the SLS can be used by both researchers and practitioners interested in studying and/or assessing the role of leadership in sustainment. One particular avenue for future research is to consider change in sustainment leadership over time, particularly in light of the dynamic nature of the sustainment process [28]. Such research could clarify whether leaders’ behaviors stabilize over time, as well as address whether consistency versus variability in leadership predicts future EBI sustainment.

Qualitative analyses also provided evidence to support the SLS. The convergence between the quantitative and qualitative results was high in that there was evidence for congruence in all four of the SLS dimensions in both methods. In addition, expansion was demonstrated in that the qualitative analysis revealed a possible fifth emergent theme: available leadership. Comments related to this theme addressed the benefits of having the leader in close proximity, being able to ask questions of the leader when needed, and leaders making time to assist with issues even when busy. This is consistent with independent work in other public sector service settings that identifies leadership availability as an implicit, but important aspect of support for EBI implementation [16]. It is important to note that available leadership has connections to other dimensions of the SLS. For instance, being available is one way in which leaders show their support for sustainment. It is also related to perseverant leadership in that the availability comments by providers were typically related to the leader being accessible and engaged when problems or issues came up, much like perseverant leadership involves the leader persevering through the challenges of sustaining an EBI. Thus, we consider the finding of this dimension as preliminary and recommend that future quantitative research evaluate whether survey items related to availability emerge as a distinct factor or dimension and the degree of overlap with items related to either supportive leadership or perseverant leadership.

Another issue identified in the qualitative analyses that deserves additional research attention concerns level of leadership. In particular, when commenting on the leader’s knowledge of the EBI, providers suggested that there were differences between the lower-level leaders’ knowledge and the knowledge of leaders further removed from points of care. The quantitative research on the ILS by Aarons et al. [20] and on the SLS in this study have focused on lower-level leaders, but more research is needed on middle- and upper-level leaders and their cross-level alignment as well [42]. It may be that different or additional behaviors are necessary at the executive level during sustainment, such as pursuing funding resources. As such, research should evaluate whether the factor structure of the SLS is consistent across leadership levels, and whether additional items are warranted depending on leadership status. In addition, it may be that different dimensions of leadership are more or less relevant at different levels. For instance, it may be the case that showing general support for the EBI is most critical for upper-level or system leaders, but that being knowledgeable about the EBI is most critical for lower-level leaders who must manage the day-to-day issues that arise with direct service providers during both implementation and sustainment.

It is important to note that the approach used here and in the development of the ILS relied on provider reports of what makes for effective leadership during implementation and sustainment, rather than a criterion-related approach. Thus, more research is needed to show the relationship between the SLS and implementation-related outcomes, such as the climate for implementation and sustainment [4, 7], employee attitudes toward EBIs [43], and ultimately, fidelity [44]. Although we did account for the nesting of the subordinates within teams and showed initial support for the aggregation of the measures at the team level, our sample size of 31 teams was not large enough to conduct multilevel confirmatory factor analysis [45]. Thus, we cannot be certain that the factor structure of the scale is the same at the group level, and thus researchers using the scale to study aggregate perceptions should do so with caution. In addition, the ICC(1) values for the measure were not as strong as we would have expected. Given the relatively high within-group agreement levels, the ICC(1) values were likely attenuated by low between-group variability in the scores. Future research with a larger number of units and higher levels of between-group variability should address both the factor structure at the unit level and the ICC(1) values for the measure. Our focus in this study was subordinate perceptions of leadership, and we acknowledge recent streams of research addressing factors associated with levels of agreement or differentiation among subordinates in their perceptions of leadership [46–48]. Future research should ensure that the level of theory for a particular study is aligned with the level of analysis for that study [49] and should specifically address possible factors that may shed light on when there is agreement about sustainment leadership within the unit and when there is not. Another avenue for future research would be to examine the nature of sustainment leadership across settings. This study was performed in child welfare service settings, but it is possible that the other dimensions could emerge in settings such as mental health, substance abuse treatment, or nursing. Finally, the congruence or incongruence between leader self-perceptions and supervisee perceptions of the leader’s sustainment leadership could also provide insights into sustainment-related outcomes [50, 51].

There are two potential limitations relating to the qualitative methods that are worth noting. The first is that the coding for this study focused on the set of questions that explicitly asked about leadership rather than the full transcript. This could have been a limitation as we did not examine whether additional responses were made about the leadership constructs, or whether we excluded additional themes that may have emerged in other parts of the focus groups. A second potential limitation is the use of focus groups rather than individual interviews. Although the majority of participants were open to discussing their leaders in a group setting, it is possible that individual interviews would have resulted in a more nuanced analysis [52].

It is useful to consider the practical uses of the SLS, particularly in light of the ILS as a related tool. We do not view the ILS and the SLS as competing measures, but instead created the SLS so that researchers and agencies would have a tool available to address perceptions of leaders and their support of the ongoing delivery of an EBI after active implementation has ended. Although we would expect that strong implementation leadership would increase the likelihood for more positive sustainment, it is also the case that with changing organizational priorities, sustainment may be less emphasized by leaders than implementation, which could counteract initial positive sustainment as time passes. Thus, interventions to improve implementation leadership should also address possible approaches for how leaders can continue to support an EBI and its sustainment over time.

## Conclusions

In conclusion, this study found evidence in support of the SLS measure for assessing sustainment leadership. The SLS is brief, practical, and extends past research on implementation leadership to assess how staff perceive leadership during the sustainment phase of the implementation process [2]. The SLS may be used to identify areas where leaders could direct more attention to their own leadership behaviors in order to increase the likelihood that EBIs are institutionalized into the normal functioning of the organization.
